# Intracellular Delivery of Hydrophilic Glycans Using Superchaotropic Clusters

**DOI:** 10.1002/anie.7747854

**Published:** 2026-08-12

**Authors:** Qiao Tang, Alexandru Ilie, Matthew I. Gibson

**Affiliations:** ^1^ Department of Chemistry University of Manchester Manchester UK; ^2^ Manchester Institute of Biotechnology University of Manchester Manchester UK

**Keywords:** cell glycan engineering, glycoRNA, metabolic oligosaccharide engineering, superchaotropic carriers, trehalose

## Abstract

The intracellular delivery of glycans is a significant challenge. Glycan delivery is typically achieved by per‐acetylation necessitating the use of the cytotoxic carrier solvent DMSO, or complex nanoscale delivery devices, to transit across the cell membrane. Here we deploy superchaotropic boron clusters to unlock solvent‐free intracellular delivery of azido‐glycans, as well as trehalose. The cluster B_12_Br_12_^2–^ delivered glycans into suspension and adherent cells, enabling successful metabolic oligosaccharide labelling and bioconjugation of glycoproteins, cell surface glycans, and also glycosylated‐RNA. This approach offers a practical and operationally simple solution to the challenge of glycan intracellular delivery and removes the cytotoxic impact of solvents.

## Graphical abstract

Superchaotropic boron clusters are demonstrated to deliver hydrophilic glycans directly into mammalian cells, removing the need for acetylation and cytotoxic solvents such as DMSO. Azido glycans for metabolic oligosaccharide engineering, and trehalose, were delivered and the successful azido‐labelling of glycoproteins, cell surface glycans, and glycoRNA were demonstrated.

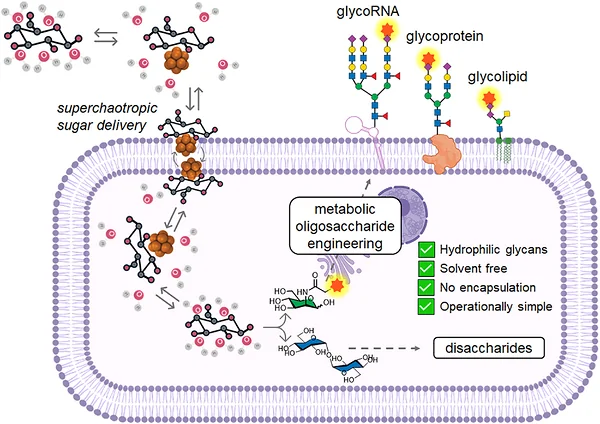

## 1 Introduction

Glycans perform a range of crucial functions in cell biology, signal transduction [1], markers of self [2], sites for pathogen engagement [3, 4] and aberrant glycosylation [5] can drive tumor progression [6]. Metabolic oligosaccharide engineering (MoE) has emerged as a versatile tool to understand, and exploit, glycan function [7, 8, 9]. In MoE an unnatural glycan, typically bearing a ‘clickable’ [10] handle is delivered to the cell, metabolized and the handle is subsequently displayed attached to the glycan. This has been deployed in basic glycobiology [11, 12, 13], cell‐surface engineering [14], targeted delivery [7, 15, 16, 17] and more. A longstanding challenge in this field, and others which require intracellular glycan delivery (such as cryobiology [18, 19]), is that most glycans have very low membrane permeability due to being hydrophilic with an extensive hydrogen bond network. This is a reason why glycans are challenging leads for drug design [20]. Electroporation [21] and ultrasound [22] have been used to deliver trehalose, but reduce cell viability and require specialist equipment. For both efficacy and practical reasons, the most widely used approach to drive passive cellular uptake of sugars is acetylation: increasing the cLogP (from −1.92 to 0.68 [23] for a mannose derivative) and exploiting intracellular esterases to ‘uncage’ the sugar [24, 25]. While simple, acetylation has the major drawback that the now insoluble sugar requires a cytotoxic carrier solvent such as DMSO: Many cell types are not tolerant to DMSO which has well characterized long‐term impacts on cell cultures, with the cryobiology field focused on reducing/replacing it [18, 26, 27, 28]. Engineered (alkylated) trehalose can enter intracellular space, for example [24], modified NeuNAc can also show increased mammalian cell uptake, but requires nontrivial chemistry and the loss of native structures [29]. As an alternative, Lee et al. loaded Ac_4_MAnNAz into vesicles for cellular delivery without solvent, but is not an operationally simple approach [15]. There are also reports that Ac_4_MAnNAz itself is cytotoxic to primary cells (neurons), necessitating alternative delivery strategies [30, 31]. Barba‐Bon et al., introduced a distinct approach to membrane translocation through the use of superchaotropic boron clusters which have high membrane affinity and passively aid cargo to transit, while being fully water soluble and showing limited cytotoxicity [32, 33, 34]. B_12_Br_12_^2−^, in particular, was found to be effective at aiding cargos up to 4.5 kDa to transit across cellular membranes, including a trisaccharide, kanamycin, on both unilamellar vesicles and Gram‐negative bacteria cells. The fast reversible binding kinetics of superchaotropic clusters to both cargoes and membrane makes it distinct from aggregation‐prone surfactants, hematolytic amphiphilic polymers [35], cationic cell penetrating peptides [36], loaded nanoparticles [37], or complex synthetic pore [38], which can cause toxicity and are not operationally simple.

Considering the above, here we introduce the use of superchaotropic boron clusters for the successful delivery of hydrophilic (no hydroxyl masking) glycan cargoes into cells, removing the need for protecting groups and cytototoxic solvents (Figure 1). Azido‐glycans are shown to enter metabolism through the successful metabolic oligosaccharide labelling of the glycocalyx, glycoproteins, and also glycoRNA. Trehalose, an impermeable disaccharide important in cryobiology [19] and autophagy [39], is also delivered.

**FIGURE 1:**
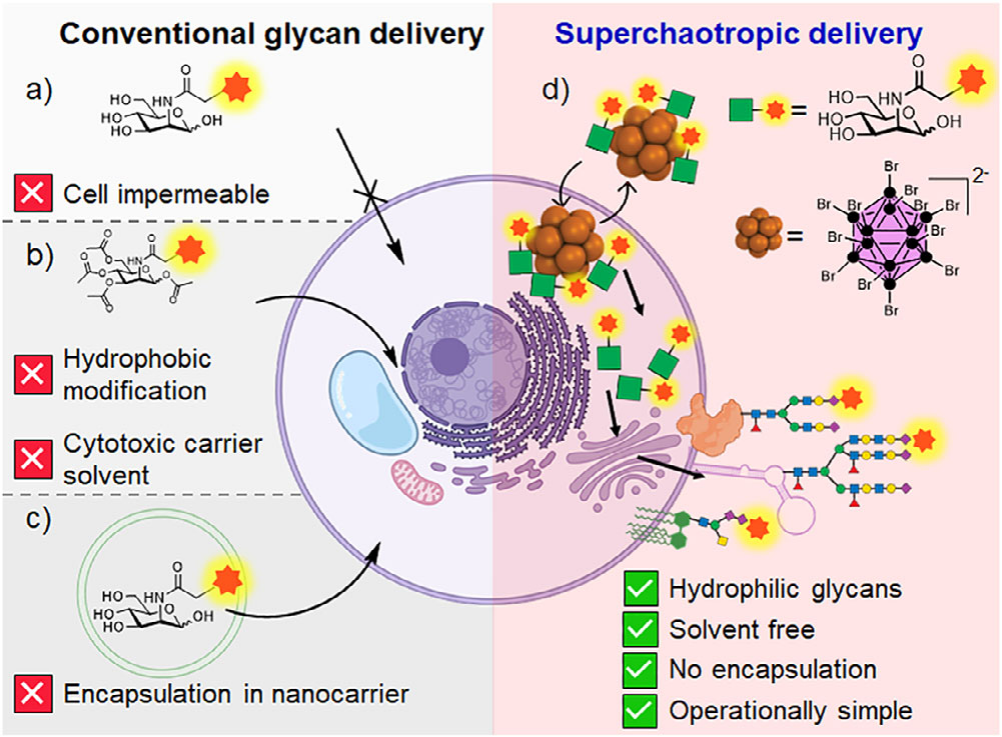
Overview of conventional glycan delivery approaches compared to super chaotropic delivery explored here. Conventional MOE re‐quires encapsulation or hydrophobization plus organic solvents. Super chaotropic boron cluster B_12_Br_12_^2−^ delivers hydrophilic cargo across cell membrane, followed by MOE of glycoproteins, glycocalyx, and glycoRNA. Created in BioRender. Tang, Q. (2026) https://BioRender.com/7hth51c.

## 2 Results and Discussion

Initial delivery experiments were conducted using ManNAz (*N‐*azido mannosamine) and the super chaotrope B_12_Br_12_^2−^ or B_12_H_12_^2−^ (which is less chaotropic [32]) as a negative control, into HeLa (human cervical cancer) cells. To remove serum effects, cells were first washed with balanced salt buffer: HEPES buffered Krebs–Ringer (HKR) solution and the ManNAz/clusters incubated with cells for 3 or 24 h. Following treatment with fluorescent DBCO‐Cy3 (to target azido‐sialic acids on the cell surface as a marker of MOE) and measurement with flow cytometry, it was seen that after 24 h there was more labelling using B_12_Br_12_^2−^, which was higher than controls (Figure S1A). While initially successful, HeLa cells with only HKR buffer for 24 h also showed significantly reduced cell viability. This was solved by using 10% Panexin CD, a serum‐free FBS replacement. As shown in Figure S1B, labelling efficiency of B_12_Br_12_^2−^/ManNAz increased by ∼30% more. With this positive data to hand, detailed labelling experiments were conducted.

Using the optimized buffer, the dose dependency of both boron clusters from 25 to 400 µM using a fixed concentration of ManNAz (25 µM) was investigated, again using DBCO‐Cy3 as the fluorescence probe and flow cytometry. As shown in Figure 2A, HeLa cells treated with increasing doses of B_12_Br_12_^2−^ lead to increased sialic acid labelling. The fraction of labelled cells in each case was higher than using B_12_H_12_^2−^ and lipofectamine 2000 (which can permeabilize cell membranes). The extent of labelling by B_12_Br_12_^2−^/ManNAz was >8 fold greater than the glycan alone, but lower than Ac_4_ManNAz, which in part could be due to eliminating the nonspecific artificial S‐glycosylation associated with per‐acetylated glycans [31](Figure S2). Using these conditions the cell viability was retained, demonstrating this to be a passive strategy (Figure S3). The dose dependency was also measured using confocal microscopy (Figure S4) showing broad agreement with the flow cytometry data that B_12_Br_12_^2−^/ManNAz labels more sialic acid than other treatments. The successful labelling was also investigated using fluorescent microscopy. Figure 2C shows merged (recolored) images with cell nucleus (blue), membrane (green), and Cy3 (red). B_12_Br_12_^2−^/ManNAz clearly leads to labelling (red) whereas B_12_H_12_^2−^had almost no detectable Cy3 labelling, indicative of enhanced delivery with the superchaotropic cluster (overview of HeLa cells under confocal microscope shown in Figure S7 and ESI videos). As a further test, we used Jurkat (human T‐cell leukemia) cells as a model suspension cell line. Using the same treatment concentrations and DBCO‐Cy3 labelling, flow cytometry showed B_12_Br_12_^2−^ labels sialic acid on Jurkat cells in a dose‐dependent manner (Figure 2B). Finally, intracellular delivery was also demonstrated using cell‐impermeable DBCO‐Cy3 and ManNAz‐Cy3 (Figures S5 and S6) that B_12_Br_12_^2−^ showing the delivered dye was distributed within the cell, but not with B_12_H_12_^2.^ This experiment was needed as the glycan visualization strategy reported metabolic labelling after delivery, rather than just measuring delivery.

**FIGURE 2:**
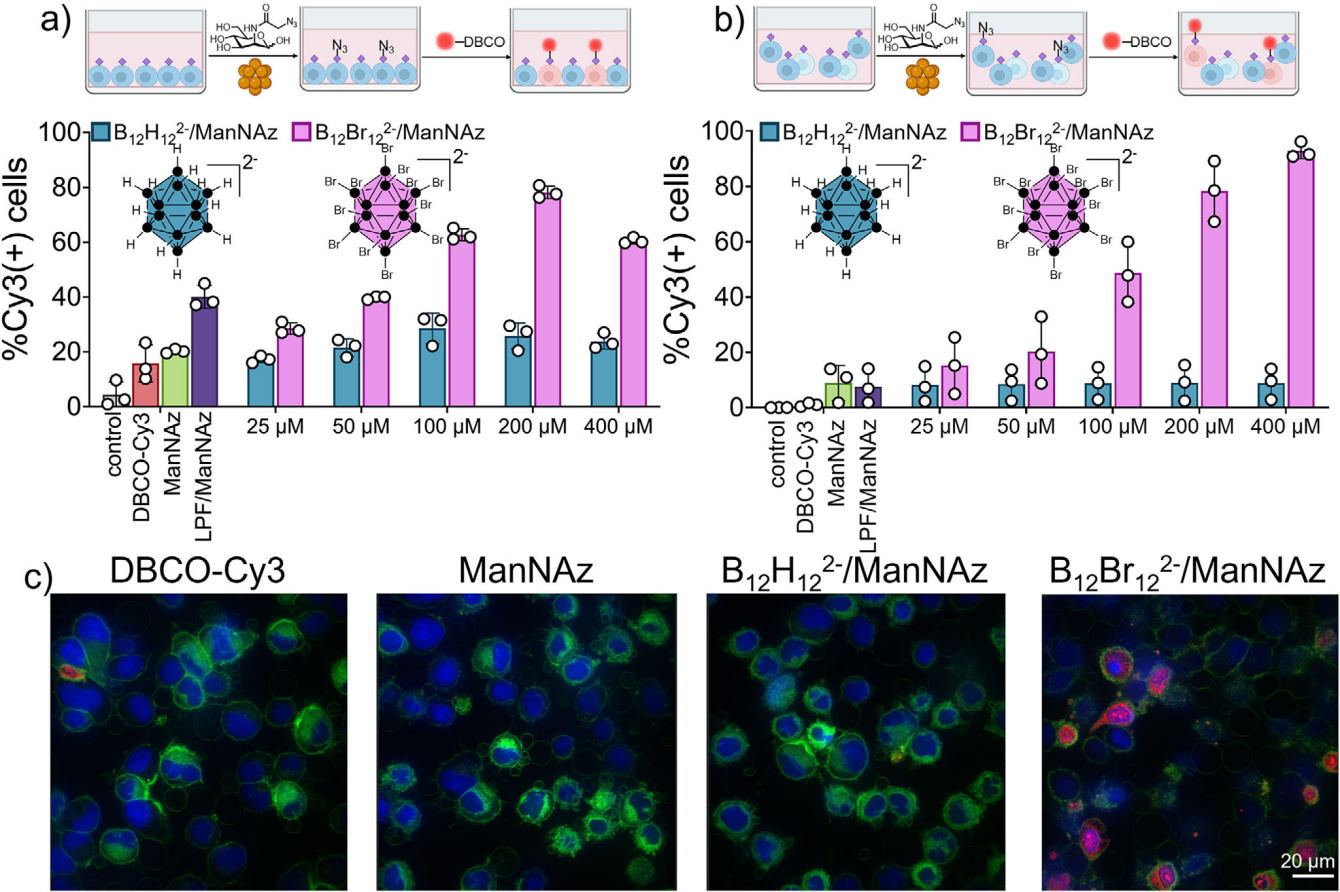
Cell‐surface sialic acid labelling by unacetylated ManNAz using super chaotropic boron clusters into: (a) HeLa and (b) Jurkat cells. Cells were incubated with clusters/ManNAz (25 µM) in HKR buffer + 10% Panexin CD for 24 h with indicated [cluster]. Cells were then washed then labelled with 25 µM DBCO‐Cy3 before analysis by flow cytometry. (c) Confocal fluorescence microscopy of HeLa cells with the indicated cluster (200 µM) and 25 µM ManNAz. Cells were washed and stained for nuclei and cell membrane before fixing with 4% formaldehyde. Hoechst 33342 for nuclei staining (blue) and CellMask plasma membrane stain (green) and DBCO‐Cy3 (red). Scale bar: 20 µm. Created in BioRender. Tang, Q. (2026) https://BioRender.com/auclh8w. Original images are listed in (Figure S4).

The above data confirmed that B_12_Br_12_^2−^ effectively delivers ManNAz, and that following metabolism the azide is presented on cell‐surface glycoconjugates. To further validate this method, we wanted to test if intracellular glycoproteins were also labelled. Using HeLa cells under the optimized delivery conditions, the cell lysate was isolated and total labelled glycoprotein was determined using SDS‐PAGE (and densiometry analysis) with DBCO‐Cy3 labelling, Figures 3A, S8, and S10. Compared to blanks, and the B_12_H_12_^2−^, B_12_Br_12_^2−^ showed significantly greater extent of labelling, validating that delivery leads to metabolism as seen with Ac_4_ManNAz/DMSO delivery methods.

**FIGURE 3:**
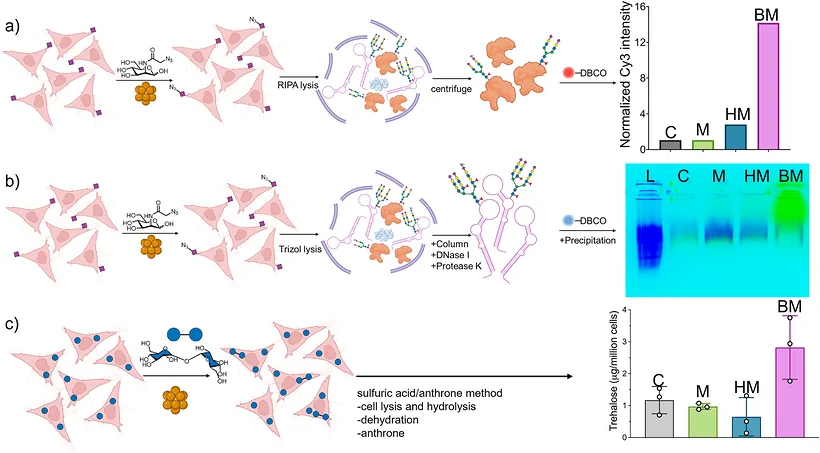
Metabolic oligosaccharide engineering by B_12_Br_12_^2−^ mediated delivery of (non acetylated) ManNAz to label intracellular glycoproteins (a) glycosylated RNA (b), and delivery of impermeable trehalose (c); (a) HeLa cells were treated with ManNAz, B_12_H_12_^2−^/ManNAz or B_12_Br_12_^2−^/ManNAz. Following lysis and isolation, the supernatant was conjugated with DBCO‐Cy3, followed by separation using SDS‐PAGE. Gels were imaged and Cy3 intensity normalized to total protein concentration; (b) Hela cells with the same treatment as (a) were washed, lysed in Trizol, purified using PureLink RNA column and then treated with turbo DNase and Protease K to remove DNA/proteins. The mixture of RNA was then cleaned up using NEB Monarch column reacted with sulfo‐DBCO‐Cy7. The conjugated GlycoRNA was extracted using Trizol reagent and precipitated using cold ethanol. The purified RNA was denatured using 18% formaldehyde and 50% glycerol containing SybrGreen II and then resolved on denatured 1% formaldehyde‐agarose gel. The gel was imaged under Cy7 channel and Sybr Green channel and the images were merged. Blue: Sybr Green II; Green: sulfo‐DBCO‐Cy7; (c); HeLa cells were treated with trehalose, B_12_H_12_^2−^/trehalose, or B_12_Br_12_^2−^/trehalose for 24 h in HKR buffer supplemented with 10% Panexin CD. Lysed cells were assayed for intracellular trehalose using CheKine Trehalose assay kit and normalized to 1 million cells. C: control; M: ManNAz; HM: B_12_H_12_^2−^/ManNAz; BM: B_12_Br_12_^2−^/ManNAz; L: ladder. Created in BioRender.Tang, Q. (2026) https://BioRender.com/0eoc5qa.

In seminal studies by Bertozzi and Flynn since 2021, small RNAs in mammalian cells have also been discovered to be glycosylated, which was previously thought not to occur [40, 41]. To further validate our delivery approach, we asked if ManNAz/ B_12_Br_12_^2−^ delivery can also label RNA‐glycoconjugates? Total RNAs from HeLa cells, treated as above, were extracted using Trizol and purified. To ensure only RNA was analyzed, the extract was treated with DNase I to first remove DNA contamination and Protease K to remove any remaining protein residues. To visualize any glycan‐azides, the near infrared fluorescent dye, sulfo‐DBCO‐Cy7, was selected to minimize any spectral overlap. Postclick, the conjugated RNAs mixture was purified by Trizol extraction and then ethanol precipitation. This step was essential as we observed that the sulfonate (from the conjugated *sulfo*DBCO‐Cy7) strongly interacts with the silica resin. The purified labelled RNAs were then denatured and stained with Sybr Green II. The denatured RNAs were then resolved on 1% formaldehyde‐agarose gel. As shown in Figure 3B, B_12_Br_12_^2−^/ManNAz treated HeLa cells (BM) showed significantly higher glycoRNA labelling signal (green) whereas all other conditions were unlabeled (only showing RNA in blue). Electrophoretic mobility of each lane was further analyzed using densitometry which shows Cy7 signal from B_12_Br_12_^2−^ lane shifted slower than other lanes. (Figure S10C). This confirms that this delivery approach is suitable for probing the emerging topic of glycoRNA.

Finally, we wanted to demonstrate that other glycans can be delivered into cells by this approach, not just those important for MOE. For this, trehalose was selected. Trehalose is not produced by mammalian cells but has widespread application in cryobiology [19], where its scope is limited by its low cell permeation and is also of relevance for mycobacterial infection [42]. Trehalose was incubated with HeLa cells using both B_12_H_12_^2−^ and B_12_Br_12_^2−^. After 24 h the total intracellular trehalose was measured by the sulfuric acid/anthrone assay. B_12_Br_12_^2−^gave a 5‐fold increase in intracellular trehalose compared to B_12_H_12_^2−^ and negative controls (Figure 3c). Although Ac_6_‐trehalose treatment is known to deliver millimolar trehalose into HeLa cells [24] it was observed that the dose of 25 µM, B_12_Br_12_^2−^ delivers more Trehalose into HeLa cells (Figure S11). It should be noted that the total delivery (3 µg/million cells) is insufficient for cryobiology, but suitable for other biochemical studies.

## 3 Conclusion

To conclude, it is demonstrated that super chaotropic boron clusters of the type B_12_Br_12_^2−^ can successfully deliver otherwise impermeable, hydrophilic, glycans into cells. This is operationally simple and requires no specialist equipment nor preparation methods. This overcomes and removes the current challenges associated with hydrophobic protecting groups, cytotoxic solvents, or multistep encapsulation/delivery vehicles while validating a conceptually new approach to glycan delivery. The delivery of ManNAz led to successful metabolic oligosaccharide labelling of cell surface glycans in both adherent and suspension cell lines, as demonstrated by flow cytometry and confocal fluorescence microscopy. Gel electrophoresis was used to demonstrate the successful labelling of intracellular glycoproteins and also glycoRNA. Intracellular delivery of trehalose was also shown to be enhanced, indicating this a versatile ‘broadband’ approach for intracellular glycan delivery. We propose this concept has broad applicably in glycobiology, cell‐surface engineering, and adjacent fields such as autophagy, cryobiology, and infection where the limited membrane permeability of glycans is a long‐standing barrier in the field. In particular this may be useful for studies of GlycoRNA which was only recently (2021 [40]) discovered. Primary cells are particularly sensitive to DMSO, or in some cases acetylated glycans, and hence this method offers a practical route to remove this toxicity. Future work will focus on increasing the extent of delivery to match the performance of acetylation, in particular through modulation of the superchaotropic carriers.

## Author Contributions

**Qiao Tang**: methodology, writing – review and editing, investigation, writing – original draft, formal analysis. **Alexandru Ilie**: investigation, methodology, writing – review and editing. **Matthew I. Gibson**: writing – original draft, writing – review and editing, project administration, resources, supervision, formal analysis.

## Conflicts of Interest

The authors declare no conflicts of interest.

## Supporting information



The Supporting Information is available free of charge on the Wiley Publications website as a PDF. This includes complete experimental details and additional data including microscopy images, cytotoxicity and gels.**Supporting File 1**: anie74152‐sup‐0001‐SuppMat.pdf.

**Supporting File 2**: anie74152‐sup‐0002‐MovieS1.mp4.

**Supporting File 3**: anie74152‐sup‐0003‐MovieS2.mp4.

**Supporting File 4**: anie74152‐sup‐0004‐MovieS3.mp4.

**Supporting File 5**: anie74152‐sup‐0005‐MovieS4.mp4.

## Data Availability

The data that supports the findings of this study are available in the Supporting Information of this article.
