# Supplementary material for: Intracellular Delivery of Hydrophilic Glycans Using Superchaotropic Clusters
Source: Angew Chem Int Ed Engl. 2026 Aug 12;65(40):e7747854. doi: 10.1002/anie.7747854 (PMC13618303; doi:10.1002/anie.7747854)
Supplement: Supplementary file 1 [file ANIE-65-e7747854-s002.pdf]

Electronic Supporting Information For

Intracellular Delivery of Hydrophilic Glycans Using Superchaotropic Clusters

Qiao Tang,[a,b] Alexandru Ilie [a,b] and Matthew I. Gibson[a,b]*

[a] Department of Chemistry, Oxford Road, University of Manchester, Manchester, UK, M13 9PL

[b] Manchester Institute of Biotechnology, University of Manchester, Manchester, UK 131 Princess Street, Manchester, M1 7DN, UK.

Physical and Analytical Methods 2
Mammalian cell culture 3
Boron cluster delivery 3
DBCO-Cy3 delivery 4
ManNAz-Cy3 delivery 5
Flow cytometry 5
Confocal microscopy 5
Resazurin assay 6
Trehalose content assay 6
In vitro labelling of glycoprotein 6
In vitro labelling of glycoRNA 7
References 17

Materials

Boron clusters ($B_{12}H_{12}^{2-}$and $B_{12}Br_{12}^{2-}$) were purchased from Katchem. *N*-azidoacetylmannosamine (ManNAz) and Ac_6Trehalose were purchased from Biosynth. Advanced RPMI 1640, Dulbecco's Modified Eagle Medium-high glucose, FBS, PSA, L-glutamine, HEPES buffered Krebs-Ringer Solution, Pierce RIPA lysis buffer, Halt™ protease inhibitor cocktail (100X), PageRuler™ Plus prestained proteinladder (26619), trypsin, TrypLE™ Express Enzyme (1X, no phenol red), Sybr Green II, PureLink™ RNA Mini Kit, Trizol reagent, Trizol LS reagent, RNase-free tube, RNase Zap, Turbo DNase I, Protease K and NuPAGE™ LDS Sample Buffer (4x) were purchased from Thermo Scientific. Diethyl pyrocarbonate (DEPC), trehalose dihydrate, mPAGE® 4-20% Bis-Tris Precast Gels and Ac_4ManNAz was purchased from Sigma-Aldrich. 37% formaldehyde was purchased from Tokyo Chemical Industry. Monarch® RNA cleanup kit was purchased from New England Biolabs. Abbkine CheKine™ Mirco Trehalose Content Assay Kit (KTB1335) was purchased from Tebubio. Fluorescent dye DBCO-Cy3 was purchased from Sigma-Aldrich and sulfo-DBCO-Cy7 was purchased from Universal Biologicals.

Physical and Analytical Methods

Cell counting. Cells were stained with trypan blue solution at final concentration of 0.1% (w/v) and then counted under Countess II Automated Cell Counter.

Fluorescent microscopy. Fluorescence microscopy of dose-dependent boron cluster treatment on HeLa cells was performed on Olympus CKX41 inverted fluorescent microscope using green filter for excitation and red filter for emission, and images were captured by CCD and saved by Cell Sense Software. Confocal microscopy was performed on Olympus IX83 Spinning disk confocal microscope using air lens for 20x magnification or oil lens for 100x magnification at room temperature. Laser intensity and exposure time for cells with different treatment was set as the same.

Flow cytometry. Flow cytometry was performed on BD Accuri™ C6 Plus flow cytometer. Cy3 signal was recorded under PE channel (FL-2).

RNA concentration measurement. RNA concentration was determined on Thermo Fisher Nanodrop.

RNA gel electrophoresis. RNA mobility was performed on Bio-Rad electrophoresis system using 1% formaldehyde-agarose gel. RNA gel was imaged using Bio-Rad ChemiDoc MP. Nuclei acid Sybr Green II channel was selected to image RNA band and Blot Cy7 channel was selected to image Cy7-conjugated glycoRNA.

Protein gel electrophoresis. Protein gel was performed on Bio-Rad electrophoresis system using commercial 4-20% Bis-Tris Precast polyacrylamide gel. Protein gel was imaged using Bio-Rad

ChemiDoc MP. Coomassie blue channel (bright field) was selected to image protein band and Blot Cy3 channel was selected to image Cy3-conjugated glycoprotein.

Protein concentration measurement. Protein concentration was determined using Thermo BCA assay kit and protein concentration curve was calibrated using BSA solutions (concentration range from 0-2 mg/mL).

Mammalian cell culture

HeLa cells were grown in 175 cm^2 Nunc cell culture flasks (Thermo Fisher, Rugby, UK) with high glucose DMEM (Gibco, Paisley, UK) supplemented with 10% non-USA origin fetal bovine serum (FBS), 100 units/mL penicillin, 100 µg/mL streptomycin, 250 ng/mL amphotericin B (PSA) (HyClone, Cramlington, UK), 1 mM sodium pyruvate and 2.5 mM L-glutamine in the presence of 5% CO_2 at 37 °C.

Jurkat cells were grown in 75 cm^2 Nunc cell culture flasks (Thermo Fisher, Rugby, UK) with advanced Roswell Park Memorial Institute (RPMI) 1640 Medium (Gibco, Paisley, UK) supplemented with 10% non-USA origin fetal bovine serum (FBS), 100 units/mL penicillin, 100 µg/mL streptomycin, and 250 ng/mL amphotericin B (PSA) (HyClone, Cramlington, UK) in the presence of 5% CO_2 at 37 °C.

Boron cluster delivery

Boron cluster was prepared using HKR buffer at 10 mM stock and sterilized using 0.22 µm filter, and the stock solution was made freshly every time performing sugar delivery. To make boron cluster/sugar mixture, sugar (ManNAz or Trehalose final concentration: 25 µM) was mixed with boron cluster ($B_{12}H_{12}^{2-}$ or $B_{12}Br_{12}^{2-}$ final concentration: 25 µM to 400 µM) in HKR buffer containing 10% Panexin CD just before delivery. To prepare delivery mixture by commercial lipofectamine 2000 (LPF2K), LPF 2K (1 mg/mL stock) was added to HKR buffer containing 10% Panexin CD and 25 µM ManNAz at final LPF2K concentration of 0.004 mg/mL and incubated at room temperature for 15-20 mins. For acetylated sugar delivery, Ac_4ManNAz or Ac_6Trehalose was dissolved in DMSO at stock concentration of 40 mM and then diluted in HKR buffer containing 10% Panexin CD at the final concentration of 25 µM (contains 0.06% DMSO).

Following standard culture conditions, HeLa cells were washed with cold HKR buffer for three times and then incubated with boron cluster/sugar mixture, sugar alone or LPF2K/sugar mixture for 24 h at 37 °C. Cells were then washed again to remove any remaining sugar before the next step experiment. For flow cytometry, treated HeLa cells were incubated with 25 µM DBCO-Cy3 for 2 h in full media and then washed again with cold DPBS before harvest by trypsin. Cells were fixed in 4% formaldehyde at room temperature for 15 mins and washed with cold DPBS for three times. Fixed cells were stored in DPBS with foil covered in the fridge and analysed on flow cytometer within a week. For fluorescent microscopy,

treated HeLa cells were incubated with 25 μM DBCO-Cy3 for 2 h and then washed again with cold DPBS before live cell imaging under Olympus CKX41 inverted fluorescent microscope. Cy3 was excited green light and emission light was monitored through red filter. Images were acquired by using Cell Sense software through a CCD connected to microscope. For confocal microscopy, treated HeLa cells incubated with 25 μM DBCO-Cy3 for additional 2 h at 37 °C. Cells were then washed again and incubated with Hoechst 33342 and Cell Mask Deep red for 10 mins. Cells were then washed and imaged for fixed cells and live cells. For fixation, cells were then washed and fixed using 4% formaldehyde solution for 15 mins at room temperature. Cells were fully washed with cold DPBS to remove any excessive formaldehyde and stored in 500 μL DPBS for confocal microscopy. Cells were stored in fridge and covered with foil and imaged within a week. For glycoprotein and glycoRNA pulling down, treated HeLa cells were harvested using RIPA lysis buffer for glycoprotein or lifted using TrypLE for glycoRNA.

Boron cluster delivery was performed similarly on Jurkat cells. Briefly, Jurkat cells were grown in 10 cm^2 petri dish at cell density of 1×10^6 /mL on the day of delivery and then washed with cold HKR buffer for three times. The cells were counted again using countess cell counter II and seeded in 96-well u-bottom plate at cell density of 2×10^6 /mL (50 μL per well). Cells were the mixed with 50 μL freshly prepared boron cluster/ManNAz, ManNAz alone or LPF2K/ManNAz and incubated for 24 h at 37 °C. Cells were then washed again by centrifuge once at 300 g for 5 mins to remove any remaining sugar. Cells were then incubated with 25 μM DBCO-Cy3 in full media for 2 h and then washed again by centrifuge at 300 g for 5 mins to remove any excessive dye. Cells were then fixed in 4% formaldehyde at room temperature for 15 mins and washed with cold DPBS for three times. Fixed cells were stored in DPBS with foil covered in the fridge and analysed on flow cytometer within a week.

DBCO-Cy3 delivery

Cell-impermeable DBCO-Cy3 was used to investigate intracellular delivery pattern of boron cluster on HeLa cells.

Following standard culture conditions, HeLa cells were washed with cold HKR buffer for three times and then incubated with boron cluster/DBCO-Cy3 mixture or DBCO-Cy3 alone in HKR buffer supplemented with 10% Panexin CD for 24 h at 37 °C. The final concentrations of DBCO-Cy3 and boron clusters are 25 μM and 200 μM, respectively. Cells were then washed again to remove any remaining dye before imaging. Treated HeLa cells were incubated with Hoechst 33342 and Cell Mask Deep red for 10 mins. Cells were then washed and imaged for fixed cells and live cells. For fixation, cells were fixed using 4% formaldehyde solution for 15 mins at room temperature. Cells were fully washed with cold DPBS to remove any excessive formaldehyde and stored in 500 μL DPBS for confocal microscopy.

ManNAz-Cy3 delivery

Cell-impermeable ManNAz-Cy3 was used to investigate intracellular delivery pattern of boron cluster on HeLa cells.

Following standard cell culture, HeLa cells were washed with cold HKR buffer for three times and then incubated with boron cluster/ManNAz-Cy3 mixture or ManNAz-Cy3 alone in HKR buffer supplemented with 10% Panexin CD for 24 h at 37 °C. The final concentrations of ManNAz-Cy3 and boron clusters are 25 μM and 200 μM, respectively. Cells were then washed again to remove any remaining dye before imaging. Treated HeLa cells were incubated with Hoechst 33342 for 10 mins. Cells were then washed and imaged for fixed cells and live cells. For fixation, cells were fixed using 4% formaldehyde solution for 15 mins at room temperature. Cells were fully washed with cold DPBS to remove any excessive formaldehyde and stored in 500 μL DPBS for confo-cal microscopy

Flow cytometry

Adherent cells HeLa were trypsinized and incubated with 4% formaldehyde solution for 15 mins at room temperature and then washed with cold DPBS for three times. Cells were resuspended in cold DPBS supplied with 10% FBS and stored on ice during flow cytometry analysis. Cells were filtered through 40 μm cell strainer before loading on flow cytometer. Fluorescence of cells were recorded under PE channel for Cy3 signal and flow cytometry data was analyzed using flow jo software. Percentage of cells with positive Cy3 signal was gated in FlowJo using DBCO-Cy3 treated HeLa cells as baseline.

Confocal microscopy

HeLa cells were pre-seeded in 4-compartments confocal chamber at cell density of 4×10^5 per mL and washed with cold HKR buffer for three times before treating with boron cluster/ManNAz or ManNAz alone for 24 h. Cells were washed with cold DPBS for three times and then incubated with 25 μM DBCO-Cy3 for additional 2 h at 37 °C. Cells were then washed again and incubated with Hoechst 33342 and Cell Mask Deep red for 10 mins. Cells were then washed and fixed using 4% formaldehyde solution for 15 mins at room temperature. Cells were fully washed with cold DPBS to remove any excessive formaldehyde and stored in 500 μL DPBS for confocal microscopy. Nuclei were imaged under DAPI channel with excitation wavelength of 345 nm and emission wavelength of 455 nm; DBCO-Cy3 conjugated sialic acid was imaged under Cy3 channel with excitation wavelength of 565 nm and emission wavelength of 550 nm; Cell membrane was imaged under 640 channel with excitation wavelength of 625 nm and emission wavelength of 670 nm. The laser intensity and exposure time for cells with different treatment was set as the same.

Resazurin assay

To assay toxicity of boron cluster delivery, resazurin assay was used to measure metabolic activity of HeLa cells with different treatment. Briefly, HeLa were pre-seeded in 96-well plate at cell density of 4 × 10^5 per mL and washed with cold HKR buffer for three times before delivery. Washed HeLa cells were then incubated with boron clusters/ManNAz, ManNAz or buffer only for 24 h in HKR buffer supplemented with 10% Panexin CD, respectively. Boron cluster dose is 25 μM to 500 μM and ManNAz dose is 25 μM. Cells were then washed with cold DPBS twice and then replaced with fresh culture media to culture for additional 24 hours before resazurin assay.

Trehalose content assay

To assay trehalose delivery on HeLa cells, we used CheKine™ Mirco Trehalose Content Assay Kit to perform the measurement. The assay kit was based on sulfuric acid/anthrone colorimetric method. Briefly, HeLa cells pre-seeded in 10 cm^2 petri dish at cell density of 8 × 10^5 per mL on the day of delivery. Cells were washed and treated with boron cluster/trehalose, trehalose alone, Ac_6Trehalose or HKR buffer (containing 10% Panexin) for 24 h at 37 °C. After 24 h, cells were washed with cold DPBS and harvest by trypsin and neutralized with full media. Cells were counted using Countess II Cell counter and centrifuged to get cell pellet. Cells were lysed using extraction buffer provided in the kit and sonicate on ice for 5 mins (20% power, ultrasonic 3 s, interval 7 s). The cell lysate was then incubated at room temperature for additional 45 mins and centrifuge at 8,000 g for 10 mins to discard the cell debris. Trehalose in the supernatant was then incubated with reagent I to be dehydrated and react with anthrone to form green complex. The mixture was heated at 95 °C for 10 mins to fully transform trehalose and the absorption of which was measured on plate reader at 620 nm. Calibration curve of trehalose standard solution was performed every time along with cell sample, with trehalose concentration range from 0.0125 to 0.2 mg/mL and deionized water was used as control. The trehalose concentration of treated HeLa cells was calculated and normalized per 1 million cells before figure plotting.

In vitro labelling of glycoprotein

Glycoprotein was resolved on 4-20% PAGE gel. HeLa cells were seeded in 10 cm^2 petri dish at cell density of 8 × 10^5 per mL on the day of delivery. HeLa cells were washed with cold HKR buffer for three times and treated with boron clusters/ManNAz, ManNAz or buffer only for 24 h in HKR buffer supplemented with 10% Panexin CD, respectively. Boron cluster dose is 200 μM and ManNAz dose is 25 μM. Cells were washed and harvested using 1.6 mL RIPA lysis buffer (containing 16 μL protease inhibitor cockatil (100x)) on ice for 10 mins. The cell lysate was collected using a cell scraper and then sonicate the pellet for 30 seconds with 50% pulse on ice. The mixture was then centrifuge at 15,000 g for 15 mins

at 4 °C and the supernatant was collected for protein analysis. The protein concentration was determined by BCA assay. Total protein solution was then incubated with 250 μM DBCO-Cy3 for 2 h at 37 °C to allow sufficient azido-glycoprotein conjugation. For running protein gel, 60 μL reaction mixture (containing around 100 μg total protein) was incubated with 20 μL loading buffer (NuPAGE™ LDS Sample Buffer (4x) premixed with 2-mercaptoethanol at 5% (v/v)) and denatured at 95 °C for 5 mins before loading on 4-20% PAGE gel. The gel was running in MES SDS Running Buffer for 160 mins at 100V to fully remove free dye from mixture (which contains significantly stronger signal than dye-conjugated glycoprotein). The gel was immediately imaged using Bio-Rad ChemiDoc MP under Blot Cy3 channel and then stained with Coomassie blue for 1h and de-stained overnight. The protein gel was imaged again using Bio-Rad ChemiDoc MP under bright field channel to quantify protein dose in each lane. Both Cy3 and Coomassie blue gel images were analysed using Fiji Image J to plot intensity of each band from each lane. The normalized Cy3 intensity was calculated by total Cy3 intensity divided by total Coomassie blue intensity.

In vitro labelling of glycoRNA

GlycoRNA was resolved on denatured RNA formaldehyde-agarose gel. Before performing RNA experiment, buffer used in this study was made in DEPC treated water (autoclaved) and bench in biosafety cabinet/fume hood was cleaned with RNase Zap to remove any RNase contamination.

To prepare 1% formaldehyde-agarose gel, 43.2mL DEPC treated water was added into 0.6 g agarose and agarose solution was boiled using microwave. 6mL of 10 x MOPS buffer (0.4 M MOPS, 0.1 M sodium acetate, 0.01 M EDTA, pH = 8, prepared in DEPC treated water) and 10.8 mL 37% formaldehyde solution were mixed thoroughly and warmed up in 50 °C water bath. The slightly cooled agarose was then mixed with warmed up MOPS and formaldehyde and poured into gel rack to completely cool down at room temperature for forming denatured RNA gel.

To obtain glycoRNA from HeLa cells, HeLa cells were treated with boron cluster/ManNAz as previously described. Briefly, HeLa were pre-seeded in T175 flask at cell density of 8×10^5 per mL on the day of delivery. HeLa cells were washed with cold HKR buffer for three times and treated with boron clusters/ManNAz, ManNAz or buffer only for 24 h at 37 °C in HKR buffer supplemented with 10% Panexin CD, respectively. The final concentration of boron cluster is 200 μM and ManNAz is 25 μM. After 24 h, cells were washed again using cold DPBS for three times to remove any remaining ManNAz in the buffer and lifted using 10mL pre-warmed TrypLE™ Express Enzyme (1X, no phenol red) for 5-8 mins at 37°C. Cells were then neutralized using 40 mL DPBS and centrifuged at 500 g for 5 mins. Cell pellet was then dissolved in 3 mL Trizol reagent on ice and stored in -80 °C overnight. Frozen cell lysate in Trizol was thawed at room temperature for 10 mins and mixed with 600 μL chloroform. By gently inverting the tube up and down for couple of times, the mixture was incubated at room temperature for additional 5 mins

before centrifuge at 12,000 g for 15 mins at 4 °C. The aqueous phase containing RNA was carefully removed from mixture and purified using PureLink RNA mini kit followed manufacture's protocol. Specifically, 2 volume of ethanol was added to aqueous RNA solution and mixed thoroughly before binding to column. The final RNA product was eluted using pre-warmed up nuclease-free water. Concentration and quality of RNA was measured on Nanodrop based on absorbance at 230 nm, 260 nm and 280 nm. The purified RNA (around 90 µL containing ~150 µg RNA) was then incubated 12 µL Turbo DNase I and 11 µL 10x DNase buffer for 30 mins at 37 °C, followed by incubation with 2 µL Protease K (20 mg/mL stock and its working concentration is 200 µg/mL) at 50 °C for 45 mins. The enzymatic digested RNA was then cleaned using NEB monarch RNA clean up kit and eluted using nuclease free water. Concentration and quality of RNA was measured on Nanodrop again before clicking with sulfo-DBCO-Cy7. In a 50 µL reaction, 40 µL RNA (around 50 µg RNA) was incubated with 10 µL sulfo-DBCO-Cy7 (stock concentration is 2.5 mM) for 30 mins at 37 °C. Reaction was then stopped by adding 200 µL nuclease-free water and then mixed 0.75 mL Trizol LS reagent followed by 200 µL chloroform. The mixtures were then mixed thoroughly and incubated for additional 5 mins at room temperature before centrifuge at 12,000 g for 15 mins at 4 °C. The aqueous phase was carefully removed, 2 M sodium chloride stock solution (final concentration 0.2 M) was added to mix thoroughly and then add 2.5 volume of pure ethanol (pre-chilled at -80°C). The mixture was then precipitated in -80 °C for 30 mins and centrifuge at 15,000 g for 15 mins. The white RNA pellet was washed with -80 °C cold pure ethanol and centrifuge again at 15,000 g for 5 mins. After discarding the supernatant, RNA pellet was dried in air for 5-8 mins and fully dissolved in 20 µL nuclease-free water. The purified RNA was mixed with 20 µL dye free loading buffer (18% formaldehyde and 50% glycerol containing 0.5 µL Sybr Green II) and denatured at 60 °C for 5 mins before loading on 1% formaldehyde-agarose gel. The gel was running in 1x MOPS buffer (diluted using DEPC-treated water) for 20 mins at 120 V and imaged using Bio-Rad ChemiDoc MP under Sybr Green channel for RNA and Blot Cy7 channel for Cy7, respectively.

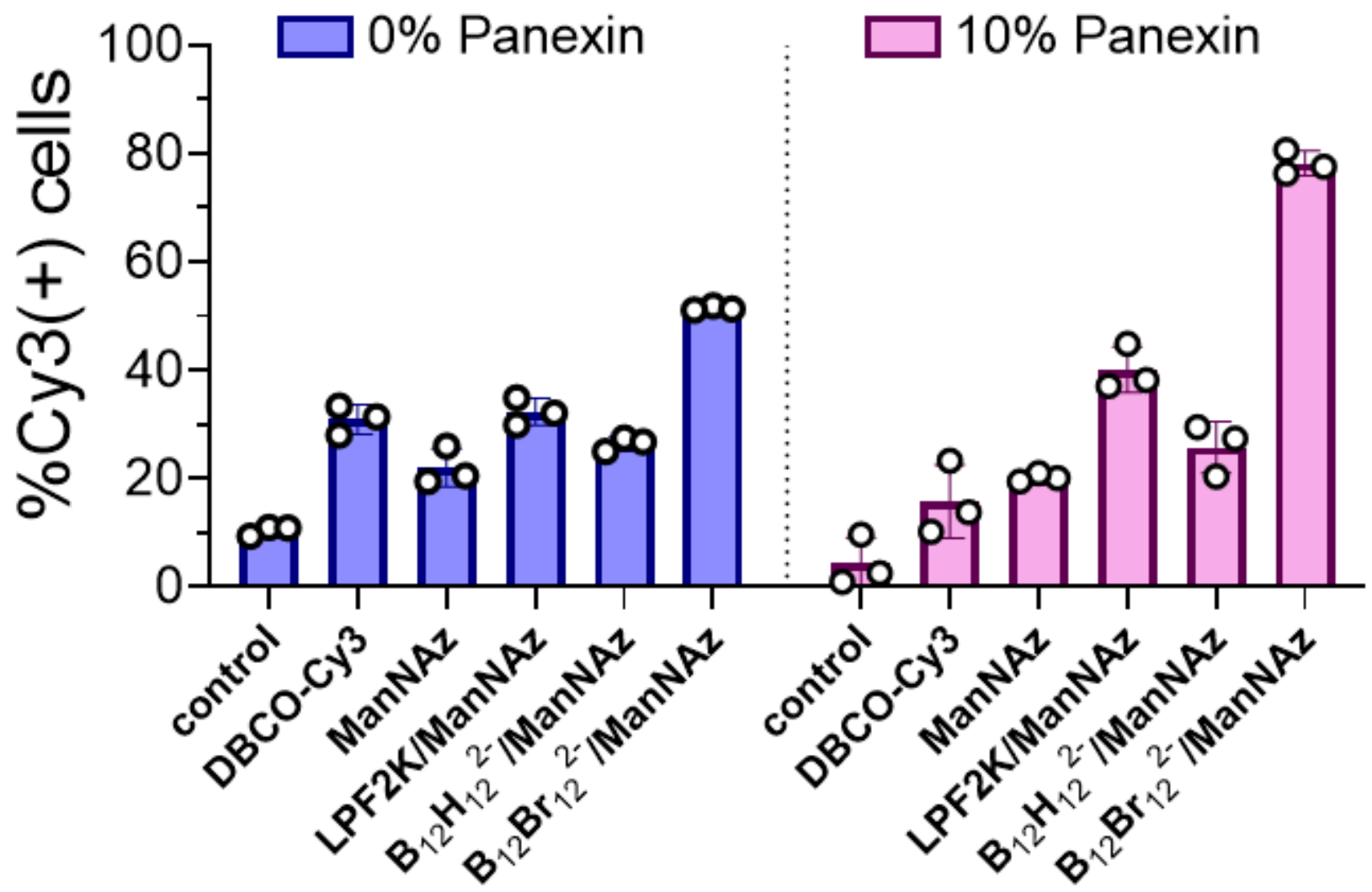

Figure S1. Effects of Panexin CD in buffer on delivery efficiency of boron cluster. HeLa cells were washed with cold HKR buffer for three times and then incubated with ManNAz, LPF2K/ManNAz, $B_{12}H_{12}^{2-}$/ManNAz, $B_{12}Br_{12}^{2-}$/ManNAz or buffer only (HKR buffer or HKR buffer supplemented with 10% Panexin CD) for 24 h. Cells were harvested and analyzed on BD Accuri C6 plus flow cytometer.

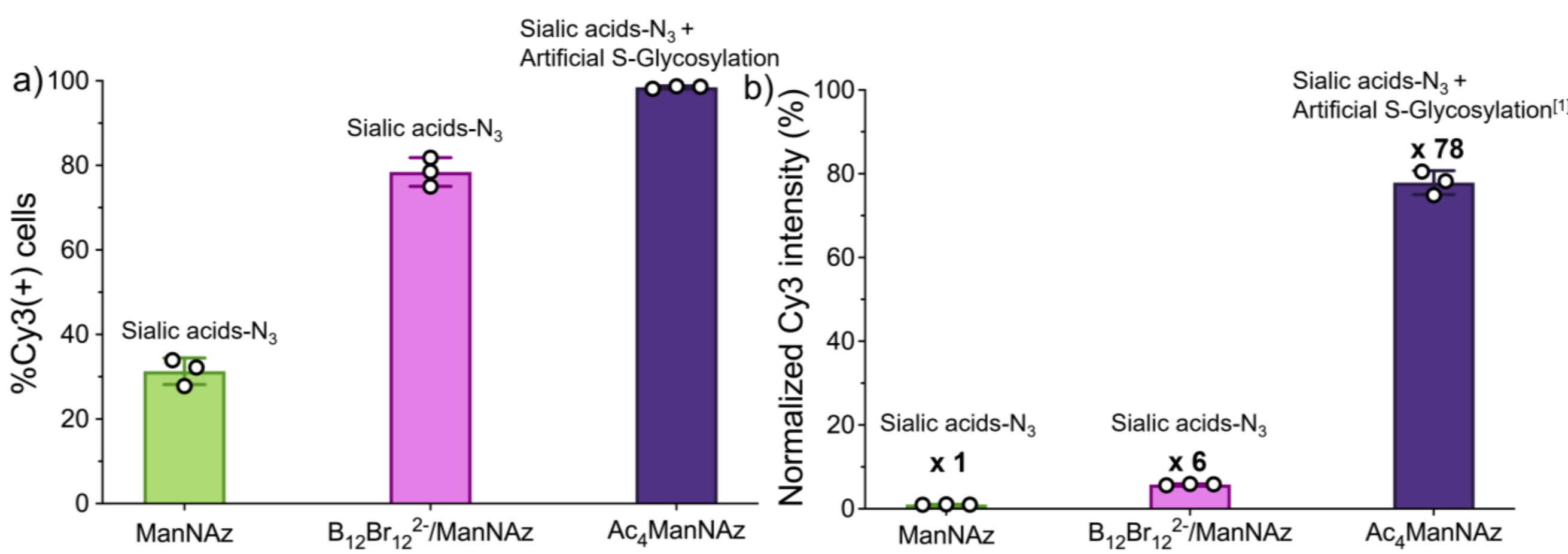

Figure S2. Comparisons of sialic acid labelling efficiency using HeLa cells. HeLa cells were washed with cold HKR buffer for three times and then incubated with ManNAz, $B_{12}Br_{12}^{2-}$/ManNAz or Ac_4ManNAz[1] in HKR buffer supplemented with 10% Panexin CD for 24 h. The final concentration of boron cluster is 200 μM and ManNAz/Ac_4ManNAz is 25 μM. Cells were harvested and analyzed on BD Accuri C6 plus flow cytometer.

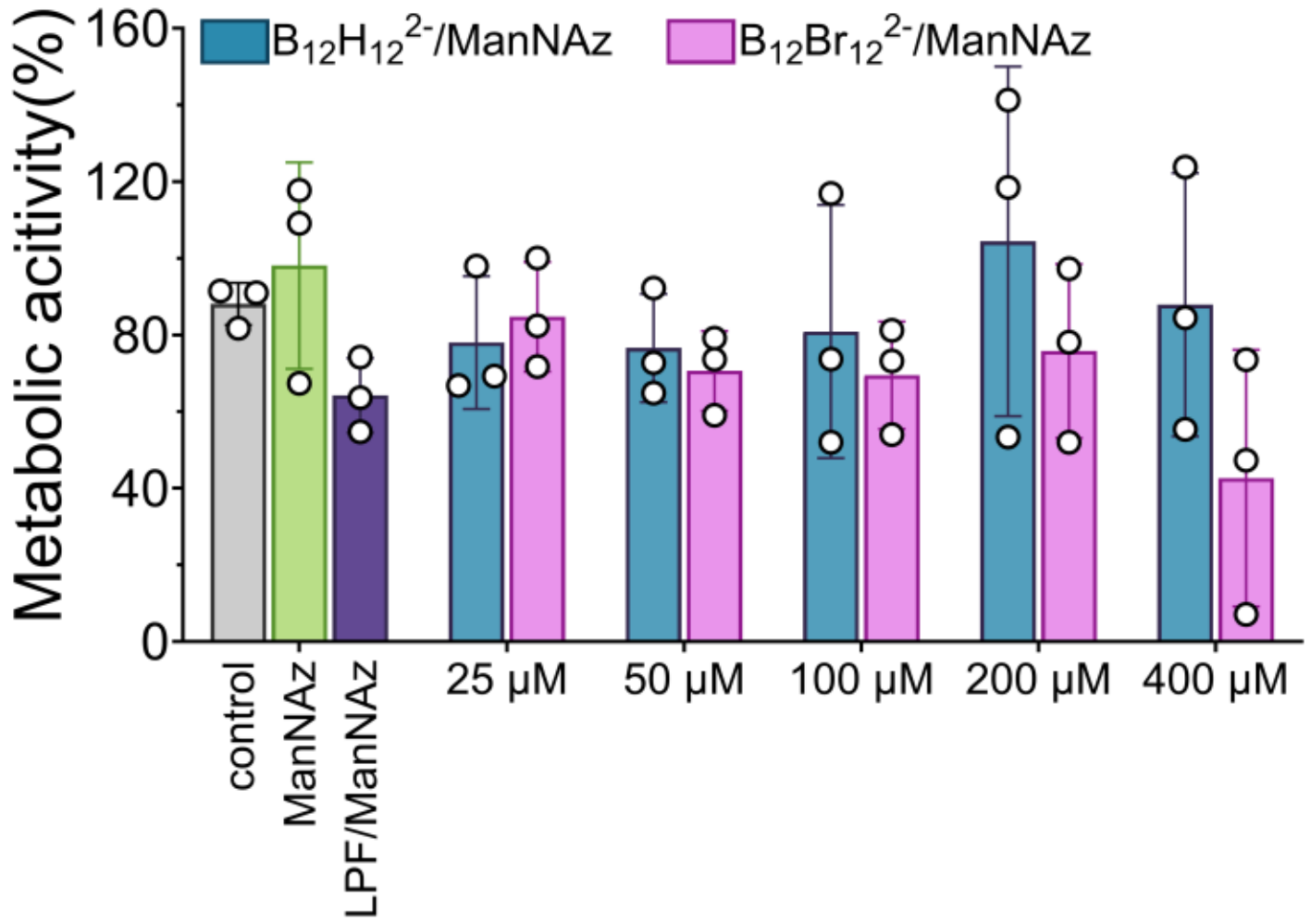

Figure S3. Cytotoxicity evaluation of boron cluster on HeLa cells. HeLa cells were washed with cold HKR buffer for three times and then treated with trehalose, $B_{12}H_{12}^{2-}$/Trehalose or $B_{12}Br_{12}^{2-}$/Trehalose containing 10% Panexin CD for 24 h. HeLa cells incubated HKR buffer with 10% Panexin CD was used as negative control. Cells were washed with cold DPBS for three times and incubated with resazurin solution to measure metabolic activity on plate reader.

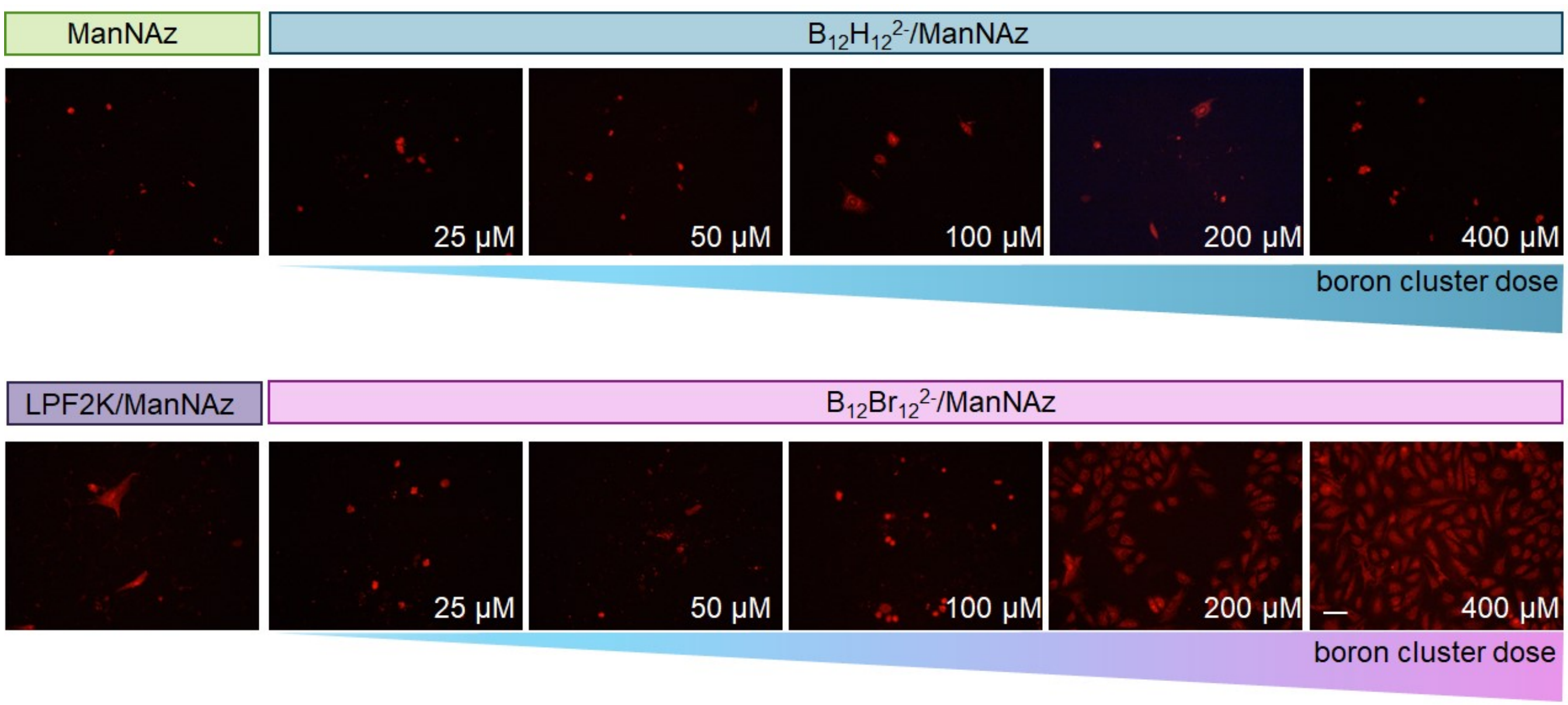

Figure S4. Fluorescence microscopy of dose-dependent boron cluster delivery on HeLa cells. HeLa cells were treated with ManNAz, $B_{12}H_{12}^{2-}$/ManNAz or $B_{12}Br_{12}^{2-}$/ManNAz as previously described and then washed with cold DPBS and incubated with DBCO-Cy3 for 2 h before imaging. Labelled glycans are in red. Final concentration of ManNAz is 25 µM and LPF is 0.004 mg/mL. Scale bar: 20 µm.

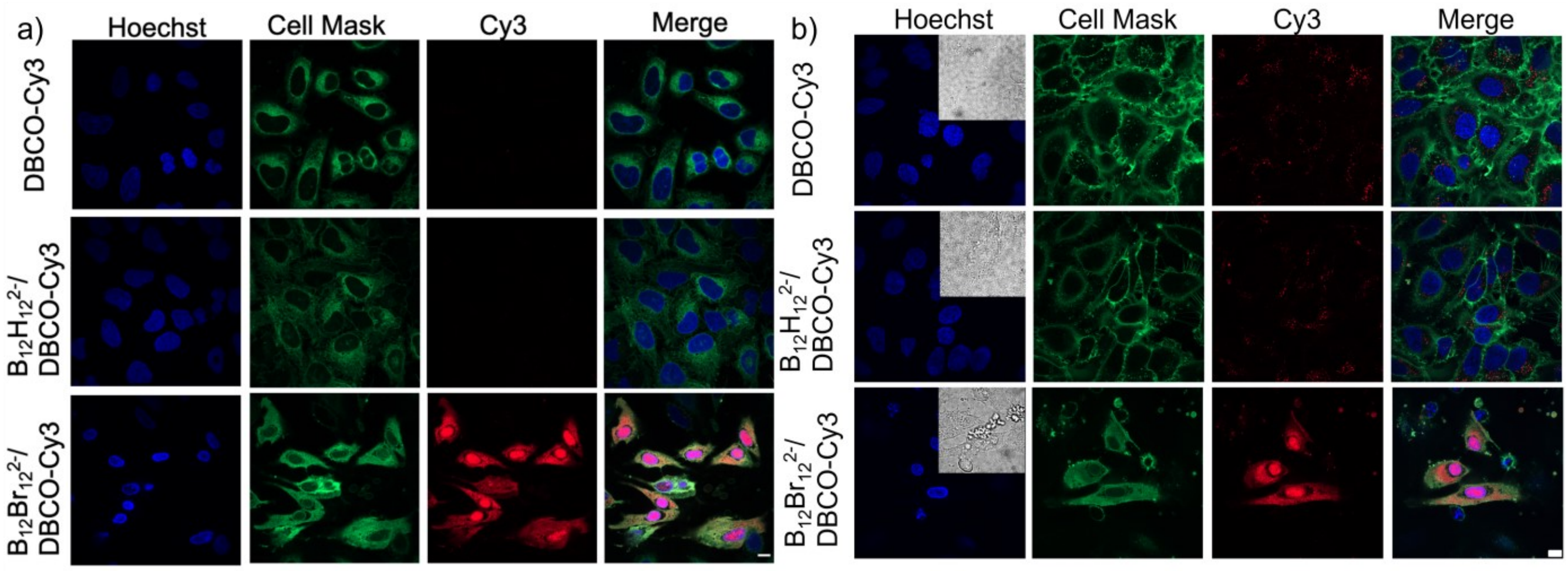

Figure S5. Confocal images of DBCO-Cy3 delivery into HeLa cells by boron clusters. HeLa cells were treated with DBCO-Cy3, $B_{12}H_{12}^{2-}$/DBCO-Cy3or $B_{12}Br_{12}^{2-}$/DBCO-Cy3 as previously described and then washed with cold DPBS for three times. Final concentration of DBCO-Cy3 is 25 μM and boron cluster is 200 μM. Cells were washed and incubated with Hoechst 33342 for nuclei staining and CellMask™ plasma membrane stain deep red for cell membrane. Cells were then washed and imaged for fixed cells (a) and live cells (b). Stained cells were imaged using Olympus confocal microscope under100x magnification using oil lens. Blue: nuclei; Red: DBCO-Cy3; Green: cell membrane; Grey: bright field. Scale bar: 10 μm for 100x magnification.

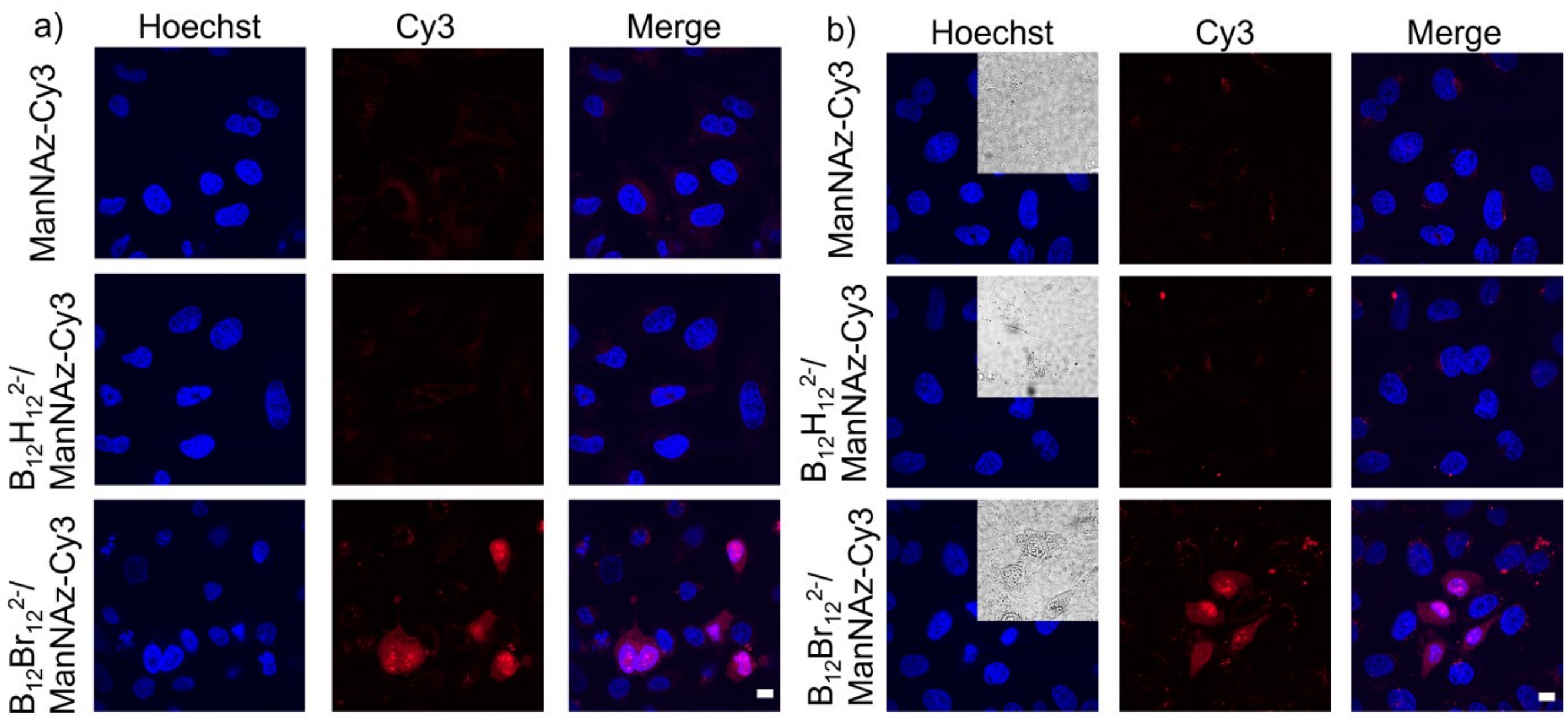

Figure S6. Confocal images of ManNAz-Cy3 delivery on HeLa cells by boron clusters. HeLa cells were treated with ManNAz-Cy3, $B_{12}H_{12}^{2-}$/ManNAz-Cy3 or $B_{12}Br_{12}^{2-}$/ManNAz-Cy3 as previously described and then washed with cold DPBS for three times. Final concentration of ManNAz-Cy3 is 25 μM and boron cluster is 200 μM. Cells were washed and incubated with Hoechst 33342 for nuclei staining. Cells were then washed and imaged for fixed cells (a) and live cells (b). Stained cells were imaged using Olympus confocal microscope under100x magnification using oil lens. Blue: nuclei; Red: ManNAz-Cy3; Grey: bright field. Scale bar: 10 μm for 100x magnification.

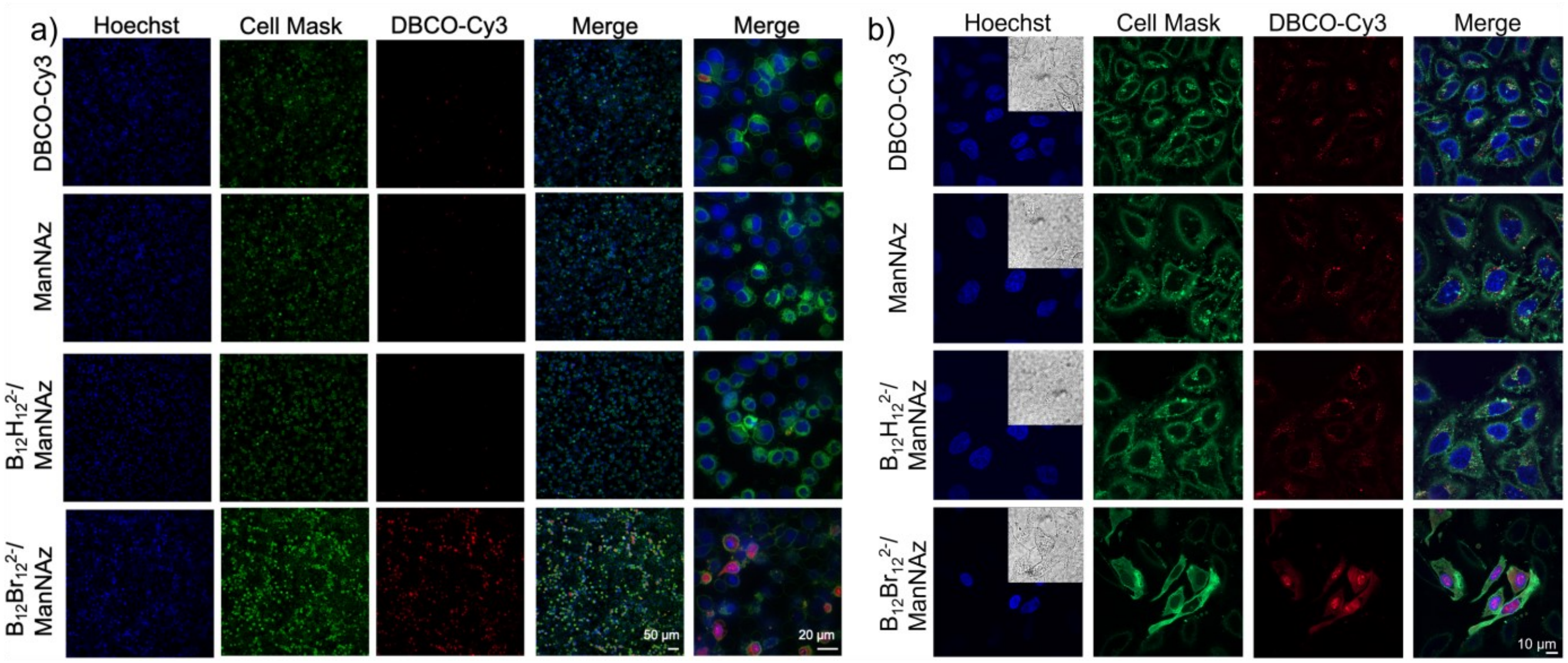

Figure S7. Confocal images of ManNAz labelled HeLa cells by boron cluster delivery. HeLa cells were treated with ManNAz, $B_{12}H_{12}^{2-}$/ManNAz or $B_{12}Br_{12}^{2-}$/ManNAz as previously described and then washed with cold DPBS and incubated with DBCO-Cy3 for 2 h. Final concentration of ManNAz is 25 μM and boron cluster is 200 μM. Cells were washed and incubated with Hoechst 33342 for nuclei staining and CellMask™ plasma membrane stain deep red for cell membrane. Cells were then washed and imaged for fixed cells (a) and live cells (b). Stained cells were imaged using Olympus confocal microscope under 20x magnification using air lens or 100x magnification using oil lens. Cells incubated with buffer only and then click with DBCO-Cy3 was used as control. Blue: nuclei; Red: glycan-Cy3; Green: cell membrane; Grey: bright field. Scale bar: 50 μm for 20x magnification and 20 μm for 100x magnification on fixed cells and 10 μm for 100x magnification on live cells.

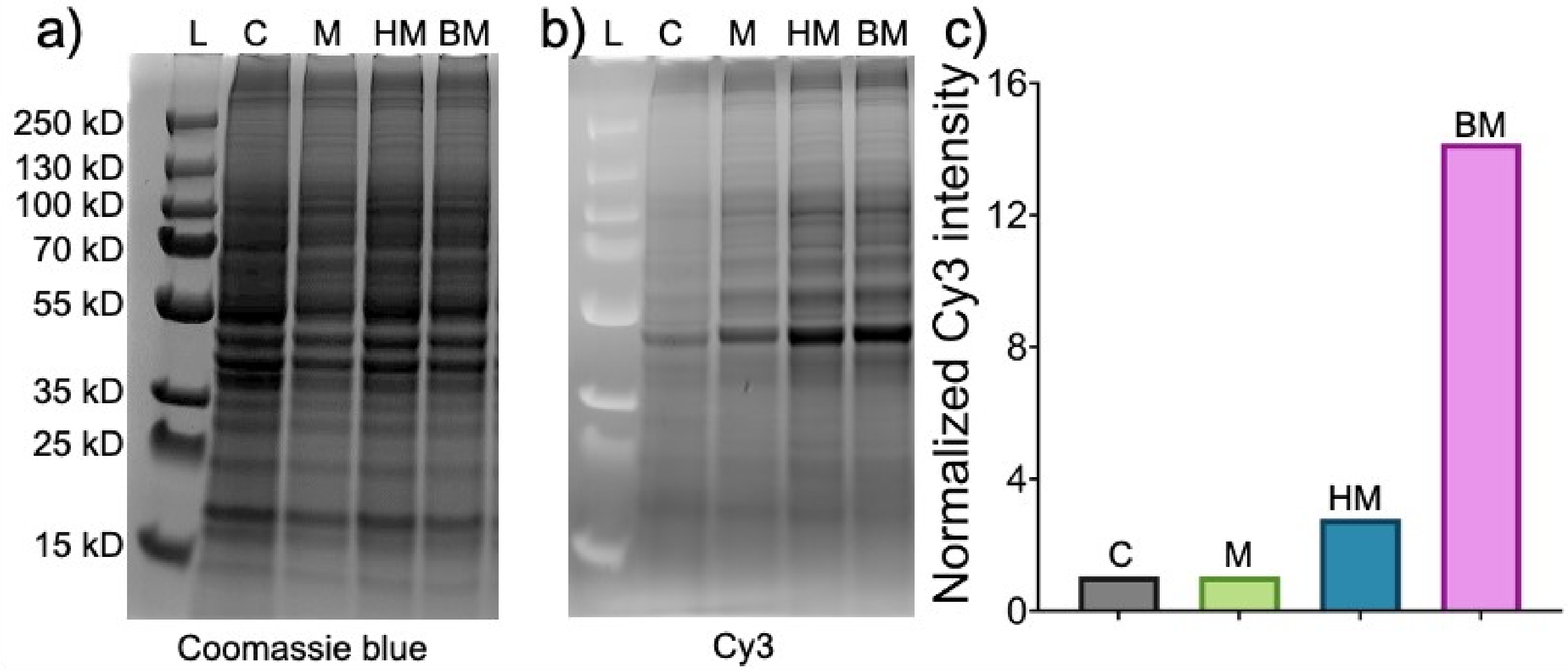

Figure S8. Glycoprotein labelling by boron cluster on HeLa cells (data in Figure 3A, repeat 1). HeLa cells were treated with ManNAz, $B_{12}H_{12}^{2-}$/ManNAz or $B_{12}Br_{12}^{2-}$/ManNAz as previously described and then washed with cold DPBS. Treated HeLa cells were harvested using RIPA lysis buffer and sonicate for 30 s before centrifuging out cell debris. Whole cell protein was clicked with 250 μM DBCO-Cy3 at 37 °C for 2 h. The mixture of protein and dye were loading on 4-20% SDS-PAGE gel and ran for 160 mins at 100 V. Gel were imaged under Cy3 channel first (b) and then stained with Coomassie blue to quantify protein amount in each lane (a) and imaged under bright field. L: protein ladder; C: control; M: ManNAz; HM: $B_{12}H_{12}^{2-}$/ManNAz; BM: $B_{12}Br_{12}^{2-}$/ManNAz.

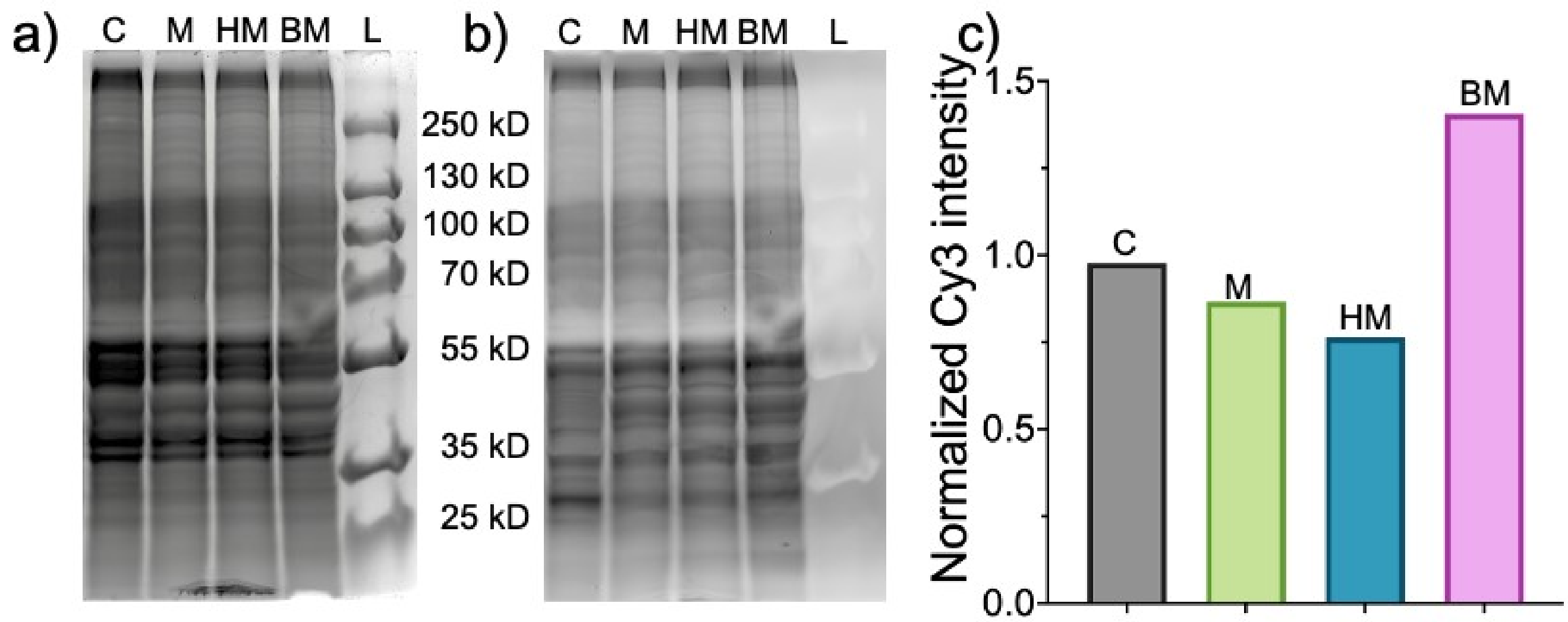

Figure S9. Glycoprotein labelling by boron cluster on HeLa cells (repeat 2). HeLa cells were treated with ManNAz, $B_{12}H_{12}^{2-}$/ManNAz or $B_{12}Br_{12}^{2-}$/ManNAz as previously described and then washed with cold DPBS. Treated HeLa cells were harvested using RIPA lysis buffer and sonicate for 30 s before centrifuging out cell debris. Whole cell protein was clicked with 250 μM DBCO-Cy3 at 37 °C for 2 h. The mixture of protein and dye were loading on 4-20% SDS-PAGE gel and ran for 160 mins at 100 V. Gel were imaged under Cy3 channel first (b) and then stained with Coomassie blue to quantify protein amount in each lane (a) and imaged under bright field. L: protein ladder; C: control; M: ManNAz; HM: $B_{12}H_{12}^{2-}$/ManNAz; BM: $B_{12}Br_{12}^{2-}$/ManNAz.

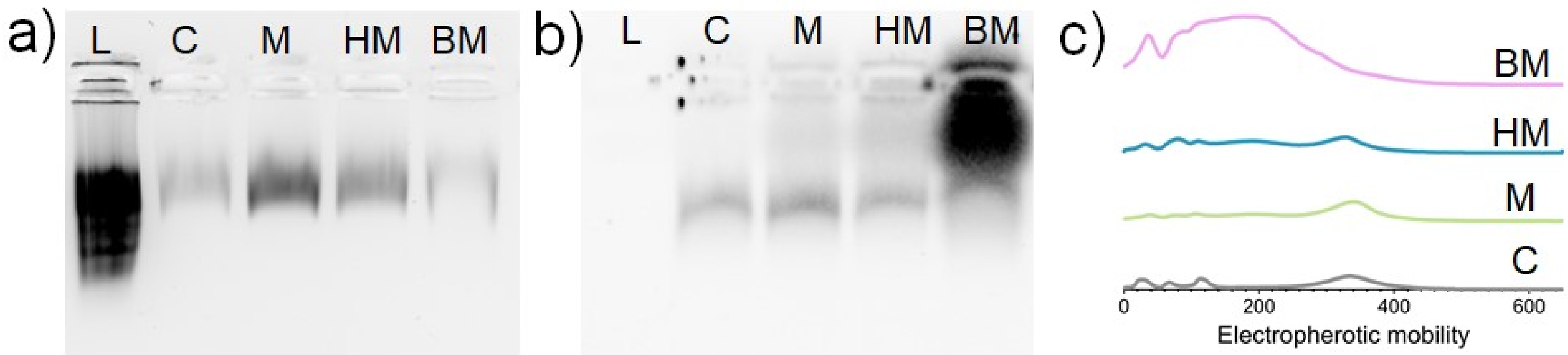

Figure S10. GlycoRNA labelling by boron cluster on HeLa cells. HeLa cells were treated with ManNAz, $B_{12}H_{12}^{2-}$/ManNAz or $B_{12}Br_{12}^{2-}$/ManNAz as previously described and then washed with cold DPBS. Treated HeLa cells were lifted using TrypLE and lysed in Trizol reagent. RNA was then extract using chloroform and purified using PureLink RNA mini kit. The purified total RNA was digested by DNase I followed by Protease K and the mixture was cleaned using NEB Monarch RNA clean up kit. The digested RNA was incubated with sulfo-DBCO-Cy7 at 37 °C for 30 mins and purified by precipitation. 20 µL of purified RNA was denatured in 20 µL dye free loading buffer (18% formaldehyde, 50% glycerol) containing 0.5 µL Sybr Green II and resolved on 1% formaldehyde-agarose gel at 120 V for 20 mins in MOPS buffer. Gel was imaged under SybrGreen II (a) for nuclei acid and Cy7 (b) for labelled glycoRNA, respectively. (c). The Cy7 gel image was further analyzed using Fiji Image J to plot electrophoretic mobility of glycoRNA-Cy7. L: dsDNA ladder 1kb plus; C: control; M: ManNAz; HM: $B_{12}H_{12}^{2-}$/ManNAz; BM: $B_{12}Br_{12}^{2-}$/ManNAz.

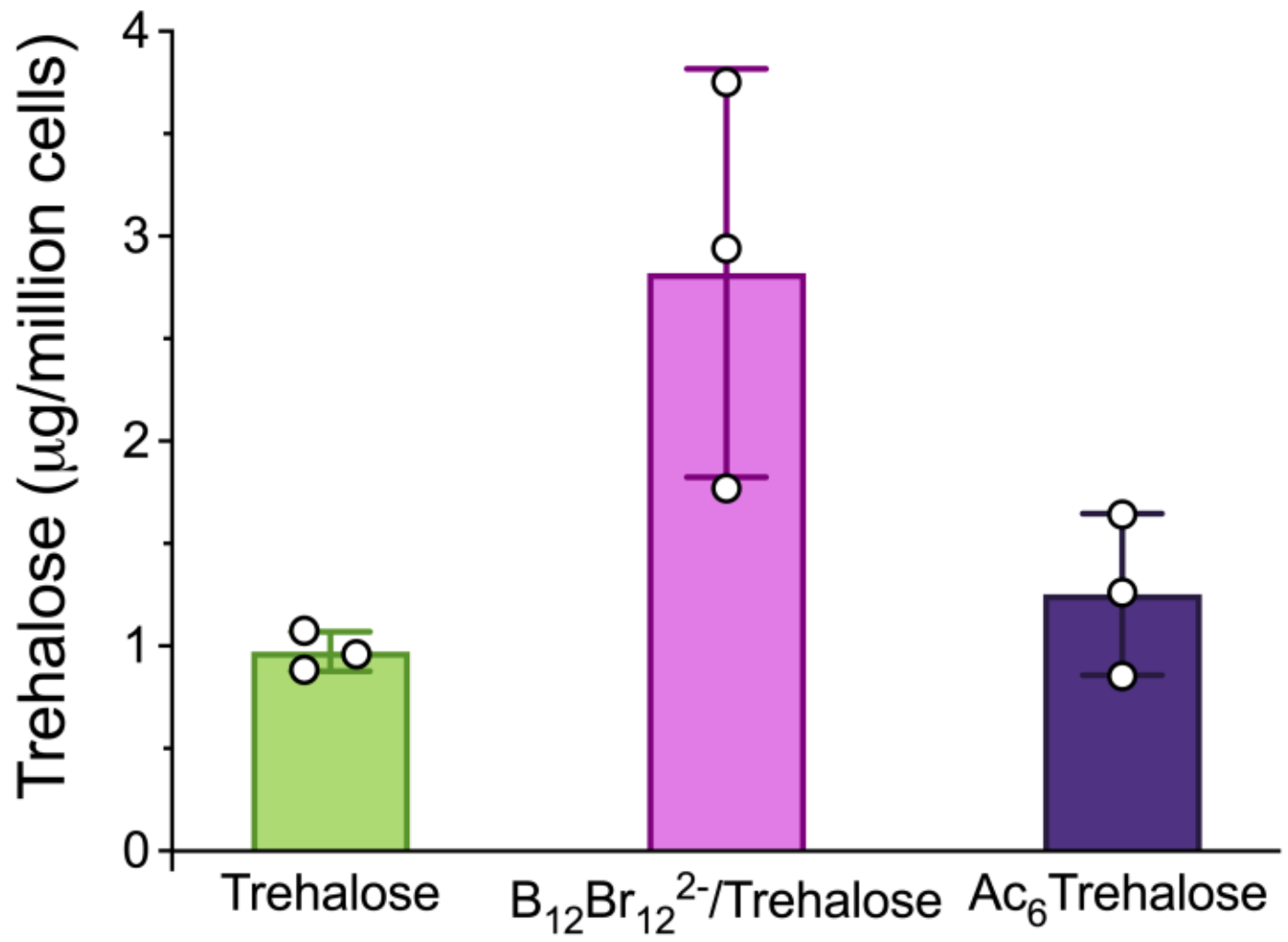

Figure S11. Comparisons of intracellular trehalose delivery efficiency on HeLa cells. HeLa cells were washed with cold HKR buffer for three times and then incubated with Trehalose only, $B_{12}Br_{12}^{2-}$/Trehalose or Ac_6Trehalose in HKR buffer supplemented with 10% Panexin CD for 24 h. The final concentration of boron cluster is 200 μM and Trehalose/Ac_6Trehalose is 25 μM. Cells were harvested and concentration of trehalose was measured using CheKine™ Mirco Trehalose Content Assay Kit.

References

[1] J. Sun, Z. Huang, Y. Du, P. Lv, X. Fan, P. Dai, X. Chen, "Metabolic Glycan Labeling in Primary Neurons Enabled by Unnatural Sugars with No S-Glyco-Modification" *ACS Chem. Biol.* **2023**, *18*, 1416–1424.
